# Supplementary material for: A UMLS-based spell checker for natural language processing in vaccine safety
Source: BMC Med Inform Decis Mak. 2007 Feb 12;7:3. doi: 10.1186/1472-6947-7-3 (PMC1805499; doi:10.1186/1472-6947-7-3)
Supplement: Additional file 13 — RAP application directory. Sets up directory for RAP (RDF application for PHP) source code [file 1472-6947-7-3-S13.gz › rap/doc/phpdoc/ontModel/OntResource.html]

xml version="1.0" encoding="iso-8859-1"?


Docs For Class OntResource


## Class OntResource

Description

Description |
Descendents
| Methods (details)

Provides a common super-type for all of the abstractions in this ontology representation package.

History: <LI>10-05-2004 : First version of this class.</LI>

- access: public
- author: Daniel Westphal <at d-westphal dot de mail at d-westphal dot de>
- version: V0.9.1

Located in /ontModel/OntResource.php (line 25)

```
Object
   |
   --Node
      |
      --Resource
         |
         --ResResource
            |
            --OntResource
```

Direct descendents

Description |
Descendents
| Methods (details)

| Class | Description |
| --- | --- |
| Individual | Interface that encapsulates an individual in an ontology, sometimes referred to as a fact or assertion. |
| OntClass | Class that represents an ontology node characterising a class description. |
| OntProperty | Class encapsulating a property in an ontology. |

Method Summary

Description |
Descendents |
Methods (details)

OntResource
OntResource
([string $uri = null])

boolean
addComment
(object ResLiteral $comment)

boolean
addIsDefinedBy
(object ResResource $resResource)

boolean
addLabelProperty
(object ResLiteral $resLiteral)

object ResResource
addProperty
(ResResource $property, ResResource/ResLiteral $object)

boolean
addRDFType
(object ResResource $resResource)

boolean
addSeeAlso
(object ResResource $resResource)

object OntClass
asClass
()

object Individual
asIndividual
()

object OntProperty
asOntProperty
()

object ResLiteral
getComment
()

object ResResource
getInstanceRdfType
()

object ResResource
getIsDefinedBy
()

object ResResource
getLabelProperty
(string $uri)

object OntClass
getPropertyValue
(object ResResource $property, [string $returnType = 'ResResource'])

object ResResource
getRDFType
()

object ResResource
getSeeAlso
()

object OntClass
getVocabulary
(string $uri)

boolean
hasComment
(object ResLiteral $resLiteral)

boolean
hasLabelProperty
(object ResLiteral $resLiteral)

boolean
hasRDFType
(object ResResource $ontClass)

boolean
hasSeeAlso
(object ResResource $resResource)

boolean
isDefinedBy
(object ResResource $resResource)

array
listComments
(string $language)

array
listIsDefinedBy
()

array
listLabelProperties
([mixed $language = false])

array
listProperty
(object ResResource $property, [string $returnType = 'OntResource'])

array
listRDFTypes
([boolean $direct = true])

array
listSeeAlso
()

boolean
removeComment
(object ResLiteral $resLiteral)

boolean
removeDefinedBy
(object ResResource $resResource)

boolean
removeLabelProperty
(object ResLiteral $resLiteral)

boolean
removeProperty
(object ResResource $property, object ResResource $value)

boolean
removeRDFType
(object ResResource $resResource)

boolean
removeSeeAlso
(object ResResource $resResource)

void
setComment
(object ResLiteral $resLiteral)

void
setInstanceRdfType
(object ResResource $resResource)

void
setIsDefinedBy
(object ResResource $resResource)

void
setLabelProperty
(object ResLiteral $resLiteral)

void
setPropertyValue
(object ResResource $property, object ResResource $value)

void
setRDFType
(object ResResource $resResource)

void
setSeeAlso
(object ResResource $resResource)

void
setVocabulary
(object OntVocabulary &$vocabulary)

Methods

Description |
Descendents |
Methods (details)

Constructor OntResource (line 51)

Constructor You can supply a uri

- access: public

OntResource

OntResource
([string $uri = null])

- string
  $uri

addComment (line 75)

Add the given comment to this resource.

- access: public

boolean

addComment
(object ResLiteral $comment)

- object ResLiteral
  $comment

addIsDefinedBy (line 98)

Add a resource that is declared to provide a definition of this resource.

- access: public

boolean

addIsDefinedBy
(object ResResource $resResource)

- object ResResource
  $resResource

addLabelProperty (line 122)

Add the given Label to this resource

- access: public

boolean

addLabelProperty
(object ResLiteral $resLiteral)

- object ResLiteral
  $resLiteral

addProperty (line 713)

Add a property to this resource.

A statement with this resource as the subject, p as the predicate and o as the object is added to the model associated with this resource. If $this->rdfType is set, an additional statement about it's type is added.

- access: public

object ResResource

addProperty
(ResResource $property, ResResource/ResLiteral $object)

- ResResource
  $property
- ResResource/ResLiteral
  $object

---

Redefinition of:

ResResource::addProperty()
:   Add a property to this resource.

addRDFType (line 147)

Add the given class as one of the rdf:type's for this resource.

- access: public

boolean

addRDFType
(object ResResource $resResource)

- object ResResource
  $resResource

addSeeAlso (line 174)

Add a resource that is declared to provided additional information about the definition of this resource.

- access: public

boolean

addSeeAlso
(object ResResource $resResource)

- object ResResource
  $resResource

asClass (line 197)

Answer a view of this resource as a class

- access: public

object OntClass

asClass
()

asIndividual (line 208)

Answer a view of this resource as an Individual

- access: public

object Individual

asIndividual
()

asOntProperty (line 219)

Answer a view of this resource as a property

- access: public

object OntProperty

asOntProperty
()

getComment (line 86)

Answer the comment string for this object. If there is more than one such resource, an arbitrary selection is made.

- return: or NULL
- access: public

object ResLiteral

getComment
()

getInstanceRdfType (line 741)

returns the rdf:type, that this distinct resource is instance of.

- access: public

object ResResource

getInstanceRdfType
()

getIsDefinedBy (line 110)

Answer a resource that is declared to provide a definition of this resource.

If there is more than one such resource, an arbitrary selection is made.

- access: public

object ResResource

getIsDefinedBy
()

getLabelProperty (line 135)

Answer the label ResLiteral for this object.

If there is more than one such resource, an arbitrary selection is made.

- access: public

object ResResource

getLabelProperty
(string $uri)

- string
  $uri

getPropertyValue (line 250)

Answer the value of a given RDF property for this resource as $returnType, or null if it doesn't have one. If there is more than one RDF statement with the given property for the current value, it is not defined which of the values will be returned.

The following return Types are supported: 'OntClass', 'OntProperty', 'Individual', and 'ResResource' Default is 'ResResource'

- access: public

object OntClass

getPropertyValue
(object ResResource $property, [string $returnType = 'ResResource'])

- object ResResource
  $property
- string
  $returnType

getRDFType (line 161)

Answer the rdf:type (ie the class) of this resource.

If there is more than one type for this resource, the return value will be one of the values, but it is not specified which one (nor that it will consistently be the same one each time).

- access: public

object ResResource

getRDFType
()

getSeeAlso (line 186)

Answer a resource that provides additional information about this resource.

If more than one such resource is defined, make an arbitrary choice.

- access: public

object ResResource

getSeeAlso
()

getVocabulary (line 232)

Answer a reference to the ontology language profile that governs the ontology model to which this ontology resource is attached.

- access: public

object OntClass

getVocabulary
(string $uri)

- string
  $uri

hasComment (line 283)

Answer true if this resource has the given comment.

- access: public

boolean

hasComment
(object ResLiteral $resLiteral)

- object ResLiteral
  $resLiteral

hasLabelProperty (line 295)

Answer true if this resource has the given label.

- access: public

boolean

hasLabelProperty
(object ResLiteral $resLiteral)

- object ResLiteral
  $resLiteral

hasRDFType (line 307)

Answer true if this resource has the given rdf:type.

- access: public

boolean

hasRDFType
(object ResResource $ontClass)

- object ResResource
  $ontClass

hasSeeAlso (line 320)

Answer true if this resource has the given resource as a source of additional information.

- access: public

boolean

hasSeeAlso
(object ResResource $resResource)

- object ResResource
  $resResource

isDefinedBy (line 332)

Answer true if this resource is defined by the given resource.

- access: public

boolean

isDefinedBy
(object ResResource $resResource)

- object ResResource
  $resResource

listComments (line 344)

Answer an array of all of the comment literals for this resource.

- access: public

array

listComments
(string $language)

- string
  $language

listIsDefinedBy (line 364)

Answer an array of all of the resources that are declared to define this resource.

- access: public

array

listIsDefinedBy
()

listLabelProperties (line 376)

Answer an array of all of the label literals for this resource, with the given language, if $language is set.

- access: public

array

listLabelProperties
([mixed $language = false])

listProperty (line 428)

Answer an array of values of a given RDF property for this resource as $returnType, or null if it doesn't have one.

The following return Types are supported: 'OntClass', 'OntProperty', 'Individual', and 'ResResource' Default is 'ResResource'

- return: of ResResources
- access: public

array

listProperty
(object ResResource $property, [string $returnType = 'OntResource'])

- object ResResource
  $property
- string
  $returnType

listRDFTypes (line 400)

Answer an array of the RDF classes to which this resource belongs.

If $direct is true, only answer those resources that are direct types of this resource, not the super-classes of the class etc.

- return: Array of ResResources
- access: public

array

listRDFTypes
([boolean $direct = true])

- boolean
  $direct

listSeeAlso (line 412)

Answer an array of all of the resources that are declared to provide addition information about this resource.

- return: Array of ResResources
- access: public

array

listSeeAlso
()

removeComment (line 464)

Remove the statement that the given ResLiteral is a comment on this resource.

Returns true, if a statement was removed

- access: public

boolean

removeComment
(object ResLiteral $resLiteral)

- object ResLiteral
  $resLiteral

removeDefinedBy (line 476)

Remove the statement that this resource is defined by the given resource.

- access: public

boolean

removeDefinedBy
(object ResResource $resResource)

- object ResResource
  $resResource

removeLabelProperty (line 489)

Remove the statement that the given ResLiteral is a label on this resource.

Returns true, if a statement was removed

- access: public

boolean

removeLabelProperty
(object ResLiteral $resLiteral)

- object ResLiteral
  $resLiteral

removeProperty (line 502)

Remove the specific property-value pair from this resource.

- access: public

boolean

removeProperty
(object ResResource $property, object ResResource $value)

- object ResResource
  $property
- object ResResource
  $value

removeRDFType (line 514)

Remove the statement that this resource is of the given RDF type.

- access: public

boolean

removeRDFType
(object ResResource $resResource)

- object ResResource
  $resResource

removeSeeAlso (line 527)

Remove the statement indicating the given resource as a source of additional information about this resource.

- access: public

boolean

removeSeeAlso
(object ResResource $resResource)

- object ResResource
  $resResource

setComment (line 539)

Assert that the given string is the comment on this resource.

Any existing statements for comment will be removed.

- access: public

void

setComment
(object ResLiteral $resLiteral)

- object ResLiteral
  $resLiteral

setInstanceRdfType (line 730)

Sets the rdf:type, that this distinct resource is instance of.

If this value is set, the ontModel will add an additional statement about this resource and the fiven rdf:type

- access: public

void

setInstanceRdfType
(object ResResource $resResource)

- object ResResource
  $resResource

setIsDefinedBy (line 551)

Assert that the given resource provides a source of definitions about this resource.

Any existing statements for isDefinedBy will be removed.

- access: public

void

setIsDefinedBy
(object ResResource $resResource)

- object ResResource
  $resResource

setLabelProperty (line 563)

Assert that the given string is the label on this resource.

Any existing statements for comment will be removed.

- access: public

void

setLabelProperty
(object ResLiteral $resLiteral)

- object ResLiteral
  $resLiteral

setPropertyValue (line 578)

Set the value of the given property of this ontology resource to the given value.

Maintains the invariant that there is at most one value of the property for a given resource, so existing property values are first removed. To add multiple properties, use addProperty.

- access: public

void

setPropertyValue
(object ResResource $property, object ResResource $value)

- object ResResource
  $property
- object ResResource
  $value

setRDFType (line 592)

Set the RDF type (ie the class) for this resource, replacing any existing rdf:type property. Any existing statements for the RDF type will first be removed.

- access: public

void

setRDFType
(object ResResource $resResource)

- object ResResource
  $resResource

setSeeAlso (line 604)

Add a resource that is declared to provided additional information about the definition of this resource

- access: public

void

setSeeAlso
(object ResResource $resResource)

- object ResResource
  $resResource

setVocabulary (line 63)

Sets the reference to the assoiated vocabulary

- access: public

void

setVocabulary
(object OntVocabulary &$vocabulary)

- object OntVocabulary
  $vocabulary

#### Inherited Methods


Inherited From ResResource

> ResResource::ResResource()  
> ResResource::addProperty()  
> ResResource::equals()  
> ResResource::getAssociatedModel()  
> ResResource::getIsAnon()  
> ResResource::getProperty()  
> ResResource::hasProperty()  
> ResResource::listProperties()  
> ResResource::removeAll()  
> ResResource::removeProperties()  
> ResResource::setAssociatedModel()  
> ResResource::setIsAnon()  
> ResResource::setURI()

Inherited From Resource

> Resource::Resource()  
> Resource::equals()  
> Resource::getLabel()  
> Resource::getLocalName()  
> Resource::getNamespace()  
> Resource::getURI()  
> Resource::toString()

Inherited From Object

> Object::toString()

Documentation generated on Thu, 7 Jul 2005 13:42:32 +0200 by phpDocumentor 1.3.0RC3
